# A Case of High-Risk Myelodysplastic Syndrome Revealed After Emergent Aortic Surgery

**DOI:** 10.7759/cureus.74771

**Published:** 2024-11-29

**Authors:** Kosuke Saku, Kazuyoshi Takagi, Masahiro Hirata, Koichi Arinaga, Eiki Tayama

**Affiliations:** 1 Division of Cardiovascular Surgery, Department of Surgery, Kurume University, Kurume, JPN

**Keywords:** cardiac surgery, cytopenia, international prognostic scoring system, myelodysplastic syndromes, pseudoaneurysm

## Abstract

The outcomes of cardiac surgery in patients with hematologic disorders are significantly worse. However, details of the clinical course of each hematologic disease remain unclear. Myelodysplastic syndrome (MDS) presents with progressive pancytopenia that has the risk of infection, hemorrhage, and transformation to acute myelogenous leukemia. A 65-year-old woman who had a history of surgery for Stanford type A acute aortic dissection presented rapidly decreasing platelets and the appearance of erythroblasts in peripheral blood. A bone marrow biopsy was performed. The day after the biopsy, an impending rupture of the pseudoaneurysm at the graft anastomosis site was found accidentally by computed tomography. Although she underwent aortic root replacement, she had recurrent thrombocytopenia and anemia, along with unexpected bleeding complications after surgery. After she was diagnosed with high-risk MDS, the management was challenging, including the decision of the indication for blood transfusion and treatments for some complications. Ultimately, she died due to infection. This report provides valuable insights into the detailed postoperative course of cardiac surgery in a patient with high-risk MDS.

## Introduction

The outcomes of cardiac surgery in patients with hematologic disorders are significantly worse [1]. However, details of the clinical course of each hematologic disease remain unclear. Myelodysplastic syndrome (MDS) is a clonal hematopathy characterized by ineffective hematopoiesis and cellular dysfunction [2]. Although patients with MDS develop progressive pancytopenia that has the risk of infection, hemorrhage, and transformation to acute myelogenous leukemia [2], surgical indications for patients with MDS are unclear. We have experienced a case who was diagnosed with MDS after emergent aortic surgery. We discuss the issues arising intra- and postoperatively in a case with MDS.

## Case presentation

**Figure 1:**
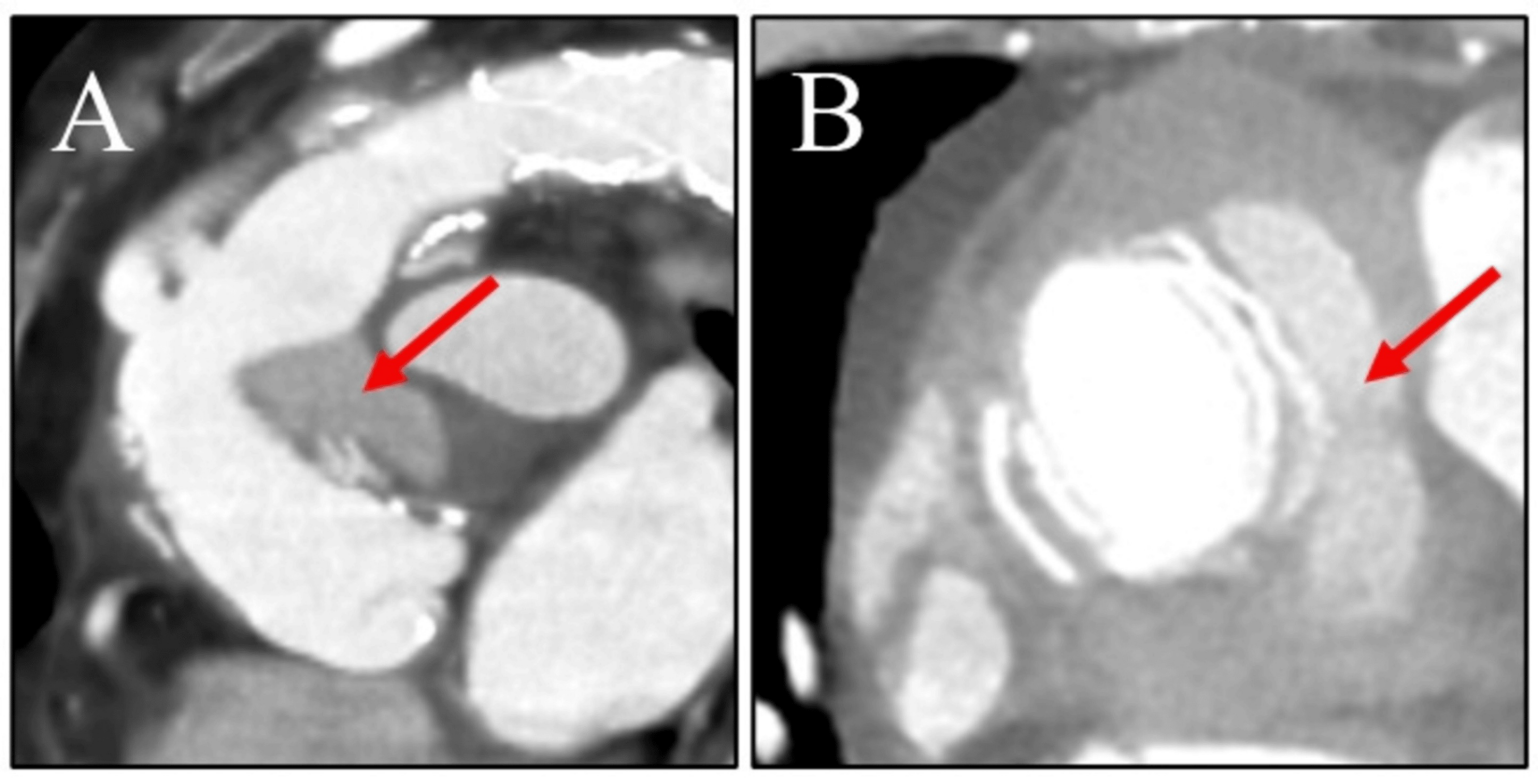
Preoperative computed tomography findings Panels A and B showed an impending rupture of the pseudoaneurysm at the graft anastomosis (arrows)

A 65-year-old woman who had a history of surgery for Stanford type A acute aortic dissection was referred to our hospital due to a rapidly decreasing hemoglobin (Hb) (7.7 g/dl) and platelet counts (2.1 × 10^4^/µL), along with the presence of erythroblasts in peripheral blood (1.5 %) (Table 1). She was taking steroids for rheumatoid arthritis. A bone marrow biopsy was carried out. The day after a biopsy, a computed tomography incidentally revealed an impending ruptured pseudoaneurysm (60 × 35 mm) at the graft anastomosis site (Figures 1A-1B). Because thrombocytopenia and the pseudoaneurysm, which had not been previously identified, were observed, we considered that blood consumption was occurring due to the aneurysm. The pseudoaneurysm has a risk of rupture; therefore, we decided to proceed with emergent surgery.

**Table 1:** Laboratory findings

Laboratory findings	Value	Reference value
Red blood cell (×10^6^ μ/L)	2.59	3.84-4.92
Hemoglobin (g/dL)	7.7	11.6-14.8
Hematocrit (%)	23.9	35.1-44.4
White blood cell (×10^3^/μL)	4.8	3.3-8.6
Neutrophil (%)	32.5	40.0-60.0
Lymphocyte (%)	32.5	26.0-46.6
Monocyte (%)	8.5	2.3-7.7
Atypical lymphocyte (%)	0.5	0.0
Erythroblast (％)	1.5	0.0
Platelet (×10^4^/μL)	2.1	15.8-34.8

However, the surgery was considered high risk since hematologic disorders were also suspected. Ultimately, we decided to perform surgery in accordance with the patient and her family’s preference. Then, aortic root replacement was performed despite the absence of a definitive diagnosis for cytopenia.

The surgical findings were as follows: significant hematoma was found around the graft. While dissecting into the pseudoaneurysm, a 10 mm ruptured site was found at the graft anastomosis. We performed aortic root replacement (Inspiris 19 mm: Edwards Lifesciences LLC, Irvine, USA, and Gelweave valsalva graft 24 mm: Terumo, Ann Arbor, MI, USA). The lowest platelet count observed during surgery was 0.5 × 10^4^/µL. Throughout the surgery, 60 units of platelets, 8 units of fresh frozen plasma, and 32 units of red blood cells were administered, resulting in hemostasis.

**Figure 2:**
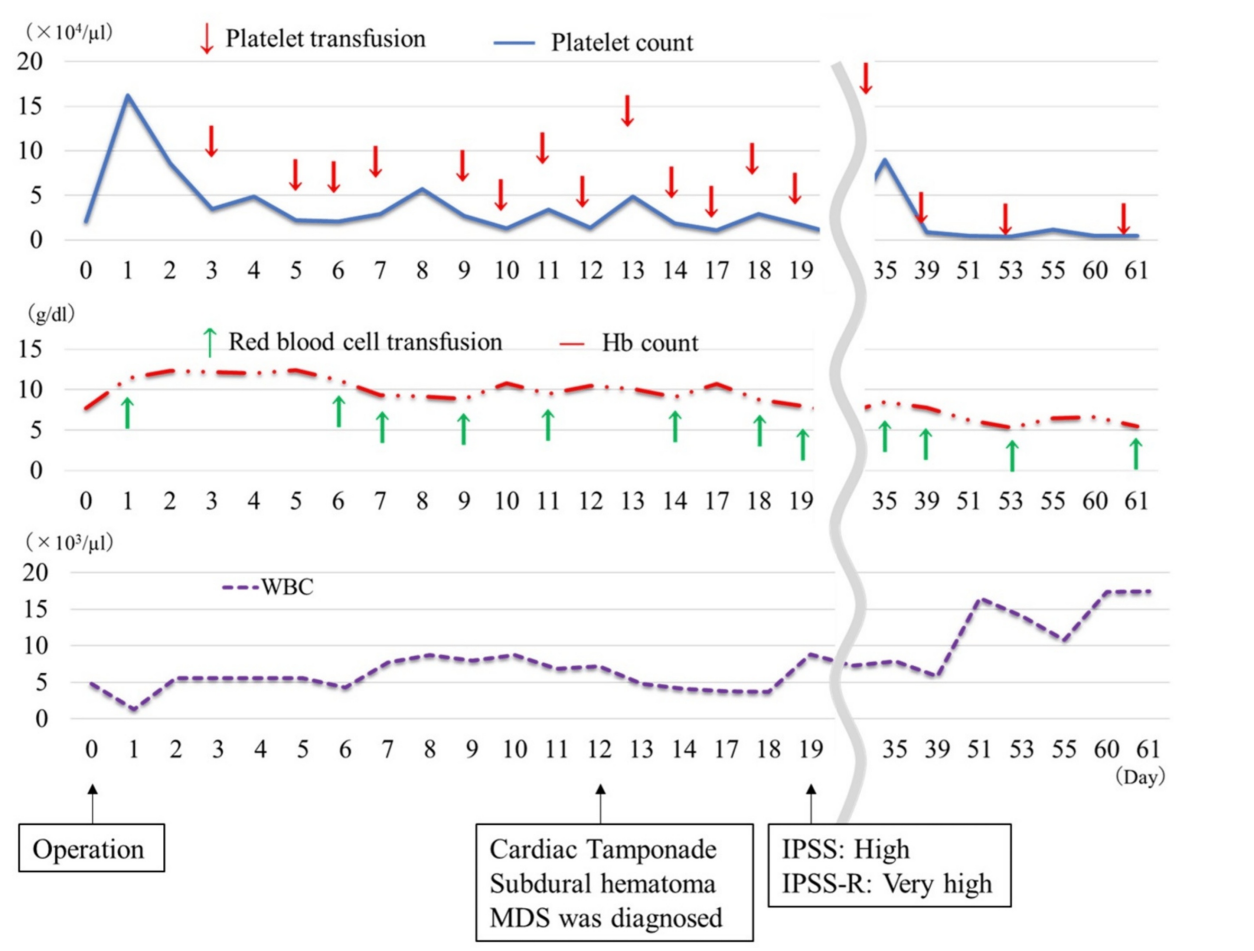
Blood cell counts during the postoperative course Arrows: transfusion; Hb: hemoglobin; WBC: white blood cell The transition of platelet, hemoglobin, and white blood cell counts during the postoperative course

**Figure 3:**
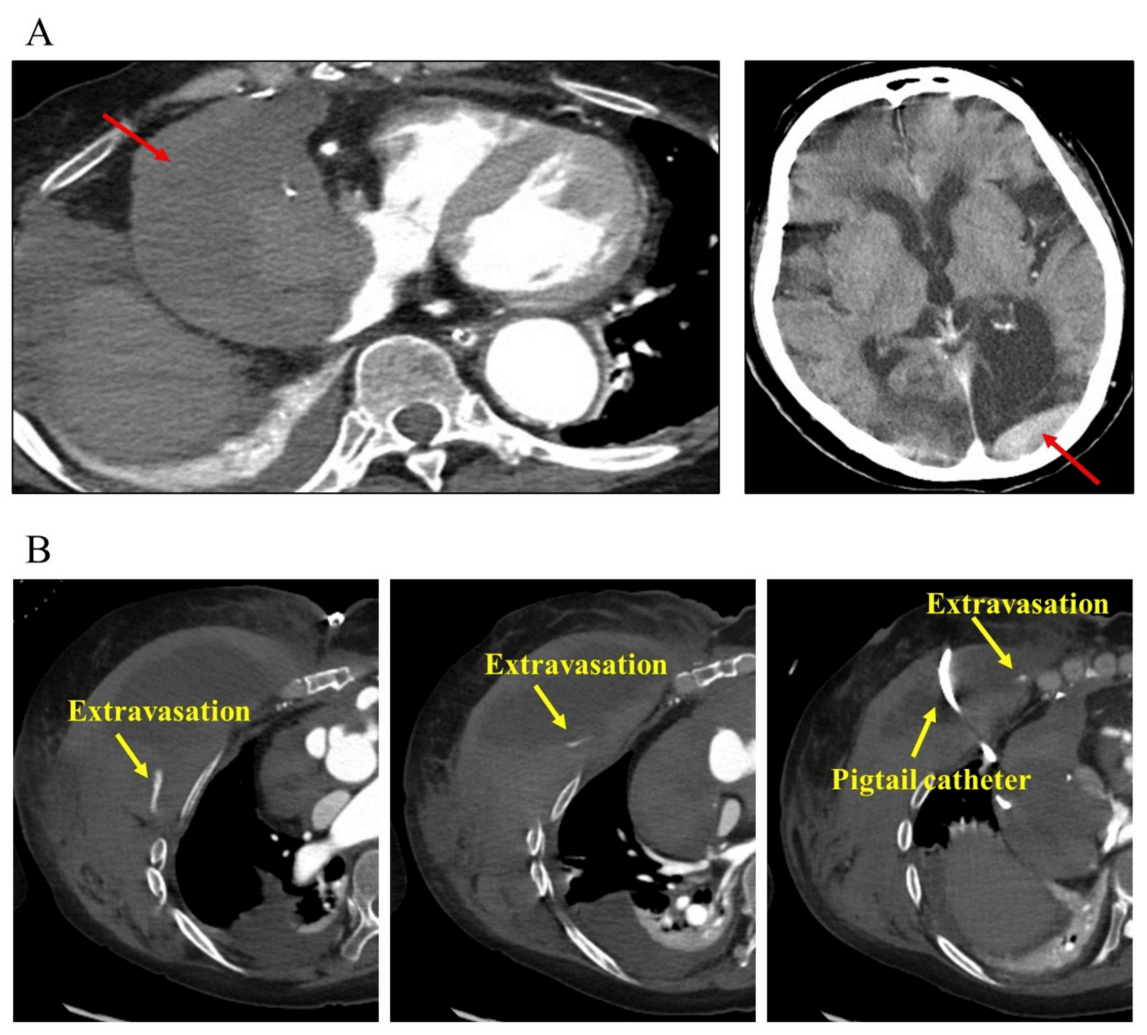
Postoperative computed tomography findings Panel A showed cardiac tamponade due to massive hematoma and subdural hematoma. Panel B showed multiple areas of bleeding within the subcutaneous hematoma

**Figure 4:**
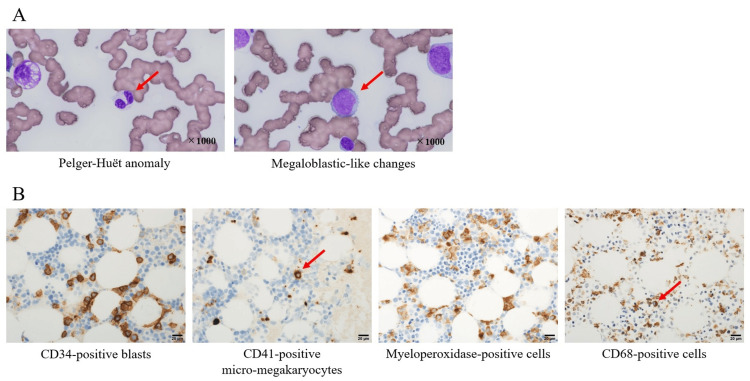
Bone marrow smear and pathological findings Panel A (bone marrow smear) showed Pelger-Huët anomaly in neutrophils and megaloblastic-like changes in erythroid precursor cells. Panel B (pathological examination) showed CD34-positive blasts, scattered CD41-positive micro-megakaryocytes, myeloperoxidase-positive cells, and CD68-positive cells

The final platelet count in the surgery was 16.0 × 10^4^/µL; however, it had rapidly decreased to 3.0 × 10^4^/µL within three days postoperatively (Figure 2). To prevent bleeding complications, daily transfusions were administered during the early postoperative period (Figure 2). However, she underwent computed tomography on the 12th postoperative day due to hemodynamic instability, revealing cardiac tamponade and subdural hematoma (Figure 3A). We performed pericardiocentesis to improve hemodynamic instability; however, unexpected bleeding occurred caused by the procedure, resulting in the formation of a subcutaneous hematoma (Figure 3B). Bone marrow biopsy revealed Pelger-Huët anomaly, and 12.2% blastlike cells were identified (Figure 4A). Additionally, pathological examination showed 10% CD34-positive blasts and scattered CD41-positive micro-megakaryocytes (Figure 4B). Immature cells positive for myeloperoxidase and CD68 were also observed (Figure 4B). Based on these findings, MDS was diagnosed after surgery. The pathological findings of the aortic wall showed no findings of connective tissue disease. In addition, on the postoperative 19th day, she was classified into International Prognostic Scoring System (IPSS): “High” and Revised IPSS (IPSS-R): “Very high” based on the bone marrow chromosomal analysis (5q deletion, seven chromosome abnormalities, and trisomy 8 were observed) and biopsy findings. There is no indication for hematopoietic stem cell transplantation and chemotherapy due to her poor condition. The management after the patient was diagnosed with MDS was challenging, including the decision of the indication for blood transfusion and treatments for some complications. There was no evidence regarding transfusion criteria; however, we aimed to transfuse to maintain Hb levels above 9.0 g/dL and platelet counts above 0.5 × 10^4^/μL. We discussed advanced care planning with her family and decided to proceed with palliative care. On the postoperative 61th day, she died of sepsis due to pneumonia. The total transfusion amount used after surgery was 230 units of platelets, 12 units of fresh frozen plasma, and 44 units of red blood cells.

## Discussion

In recent years, IPSS was developed for predicting the prognosis of MDS by scoring the percentage of bone marrow blasts, the number of cytopenic lineages, and chromosomal abnormalities [3]. An updated version called IPSS-R has been introduced, which provides more accurate prognostic predictions [4]. In this case, the patient was classified as IPSS: “High” and IPSS-R: “Very high.” The median survival reported for IPSS-R categories: “Very low,” “Low,” “Intermediate,” “High,” and “Very high” was 8.8, 5.3, 3.0, 1.6, and 0.8 years, respectively [4], indicating an extremely poor prognosis for her.

Most of the previous reports on cardiac surgery complicated with MDS involve early-stage MDS, and none of the reports included prognostic assessments [5-7]. The clinical course after surgery and complications related to high-risk MDS remain unclear [5-7]. Omoto et al. reported a case of a 67-year-old woman with MDS who underwent mitral valve repair [7]. Although the report included IPSS classification, she was categorized as low-risk and had a favorable postoperative course without any special management for MDS [7]. We have experienced a case diagnosed with high-risk MDS after emergent aortic surgery. During the course of our management, there was a significant progression of cytopenia both intra- and postoperatively, along with unexpected bleeding events. Ultimately, she died due to pneumonia.

Treatment guidelines for MDS recommend allogeneic hematopoietic stem cell transplantation or chemotherapy for high-risk groups [2]. However, allogeneic hematopoietic stem cell transplantation requires a donor and evaluation of suitability based on performance status and frailty, especially for patients over 55 years old [2]. In chemotherapy, performance status must also be considered due to potential side effects. In this case, neither allogeneic hematopoietic stem cell transplantation nor chemotherapy was deemed appropriate due to her poor conditions. Previous report suggested that the administration of granulocyte colony-stimulating factor should be considered based on the neutrophil counts [6]. In addition, minimally invasive cardiac surgery or transcatheter aortic valve implantation have been reported to be useful strategies for preventing infection and bleeding complications [5,6]. In elective surgery, these managements should be taken into consideration.

The long-term prognosis of cardiac surgery complicated with hematologic disorders has been reported to be significantly worse [1]. Especially in patients aged 60 years or older with MDS are considered to have a poor prognosis [3,4]. Therefore, in elective surgery, it is essential to adequately assess the prognosis of MDS. However, we may encounter an emergent situation like the present case; it should be necessary to establish advanced care planning and proceed to palliative care after diagnosis.

## Conclusions

We reported a case of high-risk MDS revealed after emergent aortic surgery. This report provides valuable insights into the detailed postoperative course of cardiac surgery in a patient with high-risk MDS. This case presented significant challenges in both surgery and postoperative management. We should note that the prognosis of cardiac surgery complicated with MDS is worse. When a hematologic disorder is suspected before emergent surgery, it is necessary to proceed with treatment while discussing advanced care planning with the patient and their family.
